# Influence of Crown-Implant Ratio and Implant Inclination on Marginal Bone Loss around Dental Implants Supporting Single Crowns in the Posterior Region: A Retrospective Clinical Study

**DOI:** 10.3390/jcm12093219

**Published:** 2023-04-29

**Authors:** Maha Abdul Rahim, Kashmala Khan, Bruno Ramos Chrcanovic

**Affiliations:** 1 Faculty of Odontology, Malmö University, 214 21 Malmö, Sweden; mahaa.98@outlook.com (M.A.R.); kashmala__khan@hotmail.com (K.K.); 2 Department of Prosthodontics, Faculty of Odontology, Malmö University, 214 21 Malmö, Sweden

**Keywords:** dental implants, single crown, crown-implant ratio, marginal bone loss, retrospective clinical study

## Abstract

The aim of this present record-based retrospective study was to investigate the influence of the crown-implant ratio (CIR) and implant inclination in relation to the occlusal plane on the marginal bone loss (MBL) around dental implants supporting single crowns in the posterior region of the jaws. All the cases of implant-supported single crowns in the premolar and molar regions were initially considered for inclusion. Only implants not lost, with baseline radiographs taken within 12 months after implant placement and with a minimum of 36 months of radiological follow-up, were considered for the analysis of MBL. Univariate linear regression models were used to compare MBL over time between 12 clinical covariates, after which a linear mixed-effects model was built. After the exclusion of 49 cases, a total of 316 implant-supported single crowns in 234 patients were included. The results from the statistical models suggested that implant inclination and anatomical- and clinical CIR (the main related factors investigated in the study) were not statistically significantly related to MBL over time. Age (older people), tooth region (premolar), and bruxism (bruxers) had a statistically significant influence on MBL over time.

## 1. Introduction

Vertical alveolar bone loss is a considerable issue after tooth extraction. The post-extraction alveolar bone resorption usually causes a larger coronal space and distance to the opposing jaw, also known as the interocclusal clearance. When oral rehabilitation with a dental implant is planned in sites of considerable vertical bone loss, and bone grafting is not an option, the crown-to-implant ratio (CIR) must be larger than the crown-to-root ratio for natural teeth to achieve optimal occlusal conditions [1]. This can partly affect the aesthetics of the implant-supported prosthesis and cause difficulties in future treatment with implants [2]. Moreover, the more the crown height increases, the greater the tension on the implant system will be [3,4]. The high CIR results in a greater non-axial load due to the lever arm being larger [5].

This has raised concerns about whether a high CIR would result in long-term negative effects on the marginal bone level around implants. Shorter crowns and a longer fixture length result in low CIR, which is biomechanically more favorable because it results in a shorter lever arm and thus less load on the bone; in contrast, a shorter fixture length and longer crown create an undesirable high CIR and thus an increased load on the peri-implant bone, as the lever arm becomes longer [6,7]. Some finite element analysis (FEA) studies have observed that a higher CIR in short implants increases the stress values under both axial and oblique forces, possibly having a negative effect on the maintenance of the marginal bone around implants in the long term [1,3].

From a clinical point of view, there is still no real consensus. Previous studies [8,9] showed that a high CIR creates unfavorable survival conditions and increases the risk of failure and marginal bone loss (MBL), while other studies [6] showed an inverse relationship, that rather a lower CIR results in a higher MBL.

The aim of the present retrospective study was to investigate the influence of the crown-implant ratio (CIR) and implant inclination on the MBL around dental implants supporting single crowns in the posterior region of the jaws.

## 2. Materials and Methods

### 2.1. Focused Question and Hypotheses

The focused question was elaborated by using the PICO format (Participants, Interventions, Comparisons, Outcomes): “Do CIR and implant inclination have an influence on MBL over time around implant-supported single crowns in the posterior region of the jaws”?

The first null hypothesis was that the MBL would not be affected by a greater CIR, against the alternative hypothesis that the greater the CIR is, the greater the MBL is expected to become. The second null hypothesis was that the MBL would not be affected by greater inclinations of implants in relation to the occlusal plane, against the alternative hypothesis that implants with a greater angle between the implant axis and the occlusal plane will show greater MBL.

### 2.2. Materials

This retrospective study included patients treated with dental implants during the period 1980–2018 at one specialist clinic (Clinic for Prosthodontics, Centre of Dental Specialist Care, Malmö, Sweden). This study was based on data collection from patients’ dental records. The implants were placed by specialist dentists in oral surgery, and dentists performing the prosthetic treatment were specialists in prosthodontics.

The study was approved by the regional Ethical Committee, Lund, Sweden (Dnr 2014/598; Dnr 2015/72). The present retrospective study followed the STROBE guidelines for observational studies and was registered at https://clinicaltrials.gov under the registration number NCT02369562, last updated on 3 May 2019.

### 2.3. Definitions

MBL was defined as loss, in an apical direction, of alveolar bone marginally adjacent to the dental implant, in relation to the marginal bone level initially detected after the implant was surgically placed.

**Figure 1:**
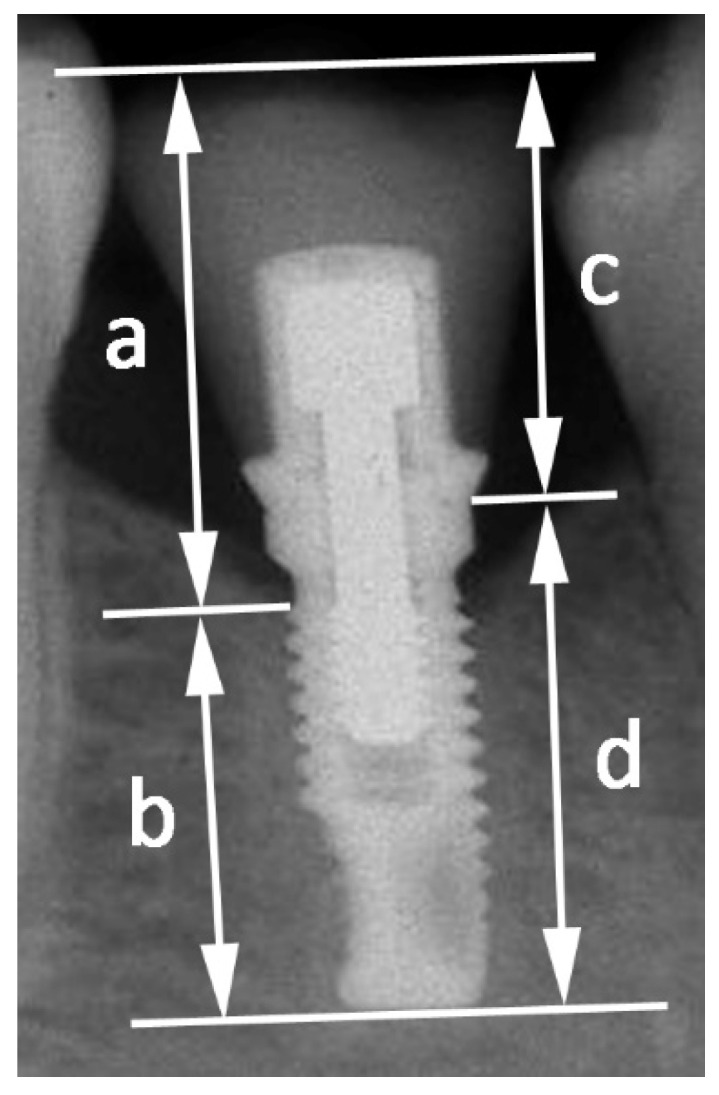
*Clinical CIR* (a/b): distance from the highest cuspid of the crown to the most coronal bone-implant contact (a) divided by the distance from the implant tip to the most coronal bone-implant contact (b). *Anatomical CIR* (c/d): distance from the highest cuspid of the crown to the crown-abutment interface (c) divided by the distance from the implant tip to the crown-abutment interface (d).

Clinical CIR (Figure 1) was referred to as the distance from the highest cuspid of the crown to the most coronal bone-implant contact divided by the distance from the implant tip to the most coronal bone-implant contact.

Anatomical CIR (Figure 1) was referred to as the distance from the highest cuspid of the crown to the crown-abutment interface divided by the distance from the implant tip to the crown-abutment interface.

**Figure 2:**
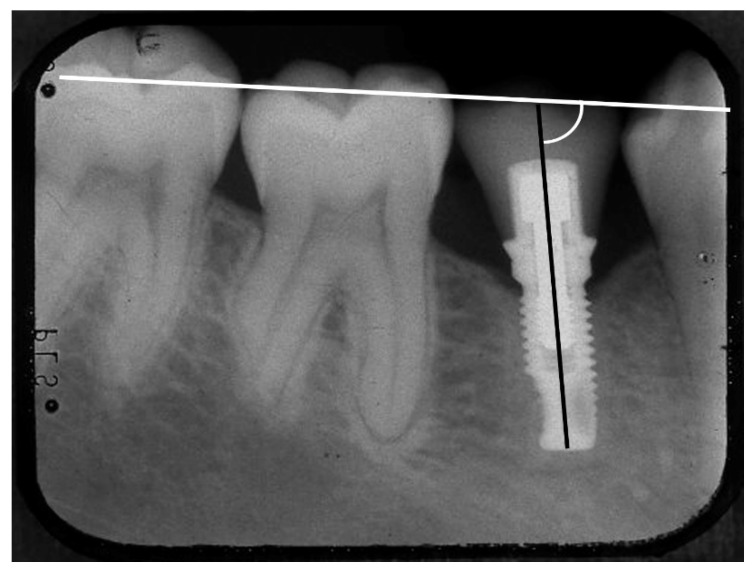
Implant inclination: the angle between the implant’s long axis (black line) and the occlusal plane (white line).

The implant inclination was defined as the angle between the implant’s long axis and the occlusal plane (Figure 2).

The authors followed the definition of bruxism proposed by an international consensus [10]: “*repetitive masticatory muscle activity characterized by clenching or grinding of the teeth and/or by bracing or thrusting of the mandible and specified as either sleep bruxism or awake bruxism*”. The sign and symptoms of bruxism were listed according to the International Classification of Sleep Disorders [11]. The diagnosis of bruxism was established according to a previous study [12], in which the patients suspected to be bruxers were called back for one clinical appointment in order to diagnose the patients as ‘probable bruxers’, based on anamnesis/self-report plus the inspection part of a clinical examination.

As standard protocol in the clinic, the patients’ dental hygiene was followed up by a dental hygienist within 6 months after the final implant-supported/retained restoration. Each patient then attended a dental hygiene recall program based on individual needs.

### 2.4. Inclusion and Exclusion Criteria

All the cases of implant-supported single crowns in the premolar and molar regions were considered.

The following inclusion/exclusion criteria were followed according to a previous study [13]. Only implants not lost, with baseline radiographs taken within 12 months after implant placement and with a minimum of 36 months of radiological follow-up, were considered for the analysis of MBL. Negative values of MBL correspond to bone loss.

Patients with all modern types of threaded implants with cylindrical or conical designs were included. Zygomatic implants were not included in the study, as well as implants detected in radiography, but without basic information about them in the patients’ files.

Patients were excluded if they had a history of periodontitis and/or were treated for periodontal disease. It is important to take note that, as standard, all patients receiving implants at the Specialist Clinic for Prosthodontics were periodontally healthy at the time of implant installation. Patients with either a history or with signs of periodontal disease were treated at the Specialist Clinic for Periodontology, where they later could or not receive dental implants, according to individual needs/indications. These patients were not included in the present study.

### 2.5. Data Collection

The data were directly entered into an SPSS file (SPSS software, version 28, SPSS Inc., Chicago, IL, USA) as the dental records of the patients were being read, and it consisted of the following variables: patient age at implant installation, patient’s sex, probable bruxism (yes/no), smoking habit (yes/no), implant location (regarding jaw (maxilla/mandible) and tooth region (premolar, molar)), implant diameter (three groups: 3.00–3.50, 3.75–4.10, and 4.30–5.00 mm), prosthesis fixation (cemented, screwed), crown material (metal ceramic, full ceramic, zirconia, metal acrylic), and follow-up time.

### 2.6. Radiological Evaluation

The evaluation of the variation of the marginal bone level over time was performed according to a previous study [14]. Reproducible intra-oral periapical radiographs were used. When there were no available digital radiographs from the baseline appointment, the analogue periapical radiographs were scanned at 1200 dpi (Epson Perfection V800 Photo Color Scanner; Nagano, Japan).

**Figure 3:**
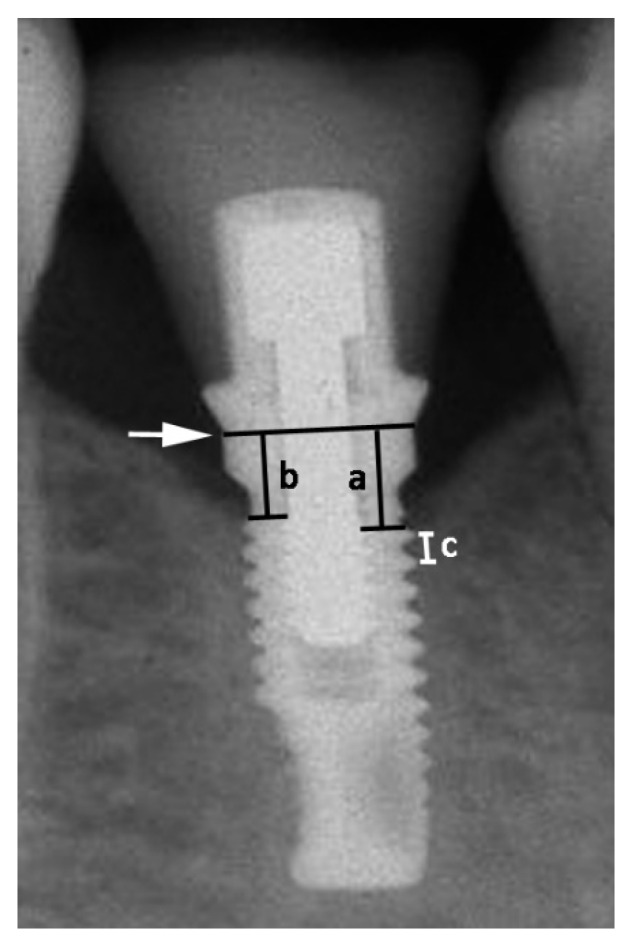
Measurement of the distance from the implant-abutment junction (black line indicated by the white arrow) to the first visible bone-to-implant contact, on both mesial (a) and distal (b) sides, on periapical radiographs. Calibration was based on the inter-thread distance of the implants (c).

MBL was measured after calibration based on the inter-thread distance of the implants. Information about the inter-thread distance was obtained from the implant catalogue of each implant manufacturer. Measurements were taken from the implant-abutment junction to the marginal bone level at both mesial and distal sides of each implant (Figure 3), and then the mean value of these two measurements was considered. MBL was calculated by comparing bone-to-implant contact levels to the radiographic baseline examination.

The two types of CIR and implant inclination were calculated according to the aforementioned definitions.

The Image J software (National Institute of Health, Bethesda, MD, USA) was used for all measurements.

### 2.7. Calibration

An initial calibration concerning MBL was performed between the authors. The process was done for 10 random samples from the cohort group and verified after the measurement of each sample. At the end of the process, the measurements from the different individuals were considered approximate enough from each other, with the agreement between examiners set at >80% of the distance in millimeters.

### 2.8. Sample Size Calculation

A calculation of the sample size was not conducted. The reason is that the database from which the eligible cases for the present study originated had a certain number of patients and dental implants, namely approximately 2800 and 11,000, respectively, and it would not be possible to recruit more cases, as the database already included all patients treated with dental implants during the period 1980–2018 in the aforementioned specialist clinic.

Instead, all cases of implants and their single crowns in the premolar and molar regions were initially considered eligible for inclusion in order to get the maximum number of cases available, namely the largest sample size possible from this database, provided that these cases would fulfill the inclusion criteria, i.e., baseline radiographs taken within 12 months after implant placement and with a minimum of 36 months of radiological follow-up.

### 2.9. Statistical Analyses

The mean, standard deviation, and percentages were presented as descriptive statistics. Kolmogorov–Smirnov test was performed to evaluate the normal distribution of the variables, and Levene’s test evaluated homoscedasticity. The tests performed for two independent groups were Student’s *t*-test or Mann—Whitney test, and for three or more independent groups, were ANOVA or Kruskal-Wallis test, depending on the normality.

Univariate linear regression models were used to compare MBL over time between clinical covariates. The estimation of MBL over time (dependent variable) was expressed in a single linear regression equation for each of the categories of each independent variable. For the present study, the linear regression equation was expressed as y = b + ax, where ‘y’ is the estimated MBL over time, ‘b’ is the estimated intercept at the y-axle in the scatter plot, ‘a’ is the estimated MBL per every one month of follow-up, and ‘x’ is the number of months of follow-up. Thus, if one would like to estimate the MBL of a certain category of a certain variable at, for example, 100 months of follow-up, ‘x’ is replaced by the value of 100 in the equation given for that particular category and variable.

In order to verify multicollinearity, a correlation matrix of all of the predictor variables was scanned to see whether there were some high correlations among the predictors. Collinearity statistics obtaining variance inflation factor (VIF) and tolerance statistics were also performed to detect more subtle forms of multicollinearity. A linear mixed-effects model was built with all variables that were moderately associated (*p* < 0.10) with MBL in the univariate linear regression models. A mixed-effects model was used in order to take into consideration that some patients had more than one implant-supported single crown, as multiple observations within an individual are not independent of each other. Multiple testing corrections for *p*-values were performed by the Bonferroni adjustment.

The degree of statistical significance was considered *p* < 0.05. Data were statistically analyzed using the Statistical Package for the Social Sciences (SPSS) version 28 software (SPSS Inc., Chicago, IL, USA).

## 3. Results

There was a total of 365 implants and their single crowns in the premolar and molar regions installed in 264 patients, among all the individuals treated with dental implants at the aforementioned specialist clinic. Of these 365 cases, there were no baseline full-implant radiographs taken within 12 months after implant placement in order to calculate clinical and anatomical CIR for 41 cases. Moreover, the periapical radiographs for the other eight cases in four patients were not of good quality. Therefore, 316 implant-supported single crowns in 234 patients were included in the present study. Most of the implants of the study were Brånemark MK implants (Nobel Biocare, Göteborg, Sweden), totaling 214 implants (69 turned/machined and 145 TiUnite implants).

The mean age (±SD) of the 234 patients was 34.9 ± 20.1 years (min-max, 15.4–90.8) on the day of the surgical implant placement. The patients were followed up clinically for a mean (±SD) of 117.8 ± 64.9 months (min-max, 18.2–361.1) and radiographically for a mean (±SD) of 104.8 ± 64.1 months (min-max, 36.1–358.7).

Table 1 shows the descriptive data of the implant-supported single crowns included in the study. The variables of patient’s age, anatomical CIR, clinical CIR, and implant inclination were divided into three categories each, based on the 33.3 and 66.7 percentiles of sample distribution, in order to generate groups of more balanced sample sizes.

The mean values (±SD) for the anatomical and clinical CIR were 0.76 ± 0.20 (min-max, 0.27–1.74) and 1.10 ± 0.31 (min-max, 0.47–2.88), respectively. The mean value (±SD) for the implant inclination was 80.4 ± 6.4 (min-max, 60–90).

**Figure 4:**
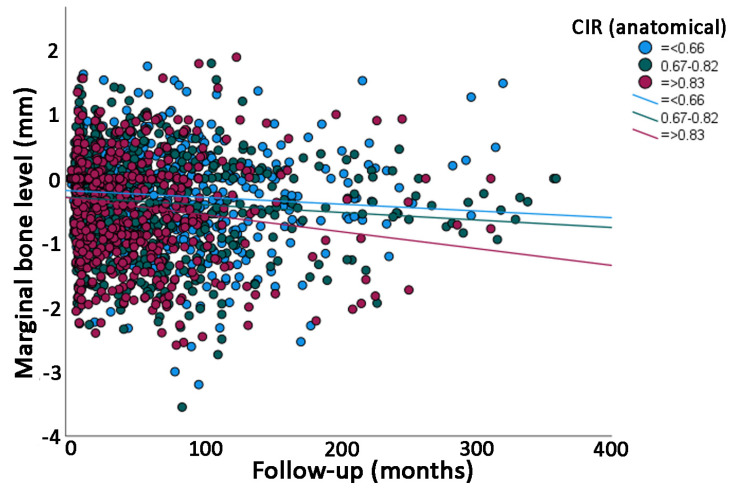
Scatter plot comparing the marginal bone level over time between groups of implants with different anatomical crown-implant ratios (CIRs) (linear regression).

**Figure 5:**
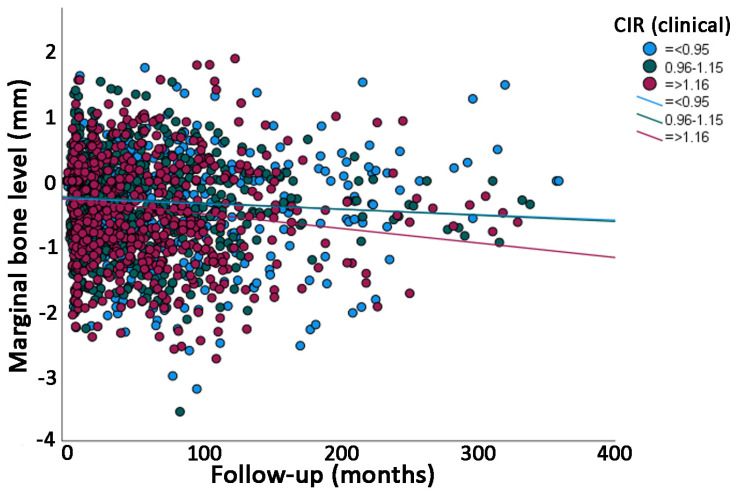
Scatter plot comparing the marginal bone level over time between groups of implants with different clinical crown-implant ratios (CIRs) (linear regression).

The univariate linear regression analysis showed that the mean loss of marginal bone over time was statistically significantly different between categories of many variables (Table 2); namely, higher in men than in women, higher in older (>40 years of age) than in younger patients, higher for implants placed in the premolar than in the molar region, higher for implant/crown sets with an anatomical CIR greater than 0.83 (Figure 4) and with a clinical CIR greater than 1.16 (Figure 5), lower in implant/crown sets with inclination lower than 78 degrees in relation to inclination with higher degrees, higher in narrow- and wide-platform implants in relation to regular-platform implants, higher in cemented than in screwed crowns, and higher in bruxers than in non-bruxers. All the categories from all the variables had a low degree of linear correlation (R^2^ linear) with MBL over time (Table 2); the highest value was 0.118 for bruxers.

The results of the linear mixed-effects model (Table 3) suggested that age (older people), tooth region (premolar), and bruxism (bruxers) had a statistically significant influence on MBL over time. Anatomical and clinical CIRs were not statistically significantly related to MBL over time.

## 4. Discussion

According to the results of the present study, there was no statistically significant difference in MBL between different groups of either anatomical or clinical CIR. This is in agreement with a recent review on the subject [15], that there was no clear correlation between either anatomical or clinical CIR and MBL, using a ratio cutoff point of 1.5 to compare groups. The present results suggested, however, an estimated greater MBL over time with greater anatomical (≥0.83) and clinical (≥1.16) CIR in relation to smaller anatomical and clinical CIR, respectively. Therefore, it can be suggested that the first null hypothesis was partly rejected.

A prospective study [9] has shown that the effects of CIR are affected by the choice of measurement. A higher clinical CIR was shown to negatively affect the prognosis of implants, whereas the differences in anatomical CIR were statistically insignificant. The authors used a total of six groups divided into anatomical- and clinical CIR with the ratios being divided between >1.50, 2.00–1.50 and >2.00, and concluded that the critical threshold values for anatomical- and clinical CIR were 3.10 and 3.40, respectively. Moreover, it is very important to point out that the thresholds for CIR suggested by this previous study of 2014 were much higher than the CIR observed in the present study. Actually, none of the implant-supported single crowns of the present study presented such high CIR as the ones for this previous study. This could suggest that a CIR even higher than the ones observed in the present study is necessary in order to possibly show a statistically significant association with MBL. However, as seen in Figure 4 and Figure 5, it was shown that the higher the CIR, the higher the estimated MBL over time. Nevertheless, the amount of estimated MBL over time for the groups of higher CIR may still not be clinically significant.

The second null hypothesis was rejected, as the difference in implant inclination in relation to the occlusal plane did not clearly affect the MBL over time. These results go against the results of an in vitro biomechanical and finite element analysis (FEA) study [16] that investigated the influence of three different implant inclinations on the microstrain distribution generated around implants submitted to an axial load of 300 N; stress was concentrated in the region between the implant and the cortical bone. The strain increased in this region with increasing implant angulation. They found a considerable difference in generated strain in the peri-implant region of implants with an angulation of 0° and 17° compared to implants with an angulation of 30°, with the latter generating higher strain. Other FEA studies also reported accentuated stresses around implants with a high degree of inclination [17,18,19]. However, the present results are in agreement with the results of a clinical study [20], which observed that implant tilting did not correlate with MBL. A review on the subject [21], gathering together data from 44 clinical studies, concluded that differences in angulation of dental implants might not affect MBL. However, it is important to point out that in the majority of these clinical studies, these angulated implants were splinted to other implants, which may bring a biomechanical advantage [22], reducing stresses at the interface between the implant and the surrounding bone [23]. The fact that the present results did not show a clear correlation between inclination and MBL in implants supporting single crowns, which are not splinted to other implants, may indicate that other factors may overcome the negative effects of implant tilting.

The present results suggested that other covariates were also associated with an estimated higher MBL with time. These factors were the patients’ age, bruxism, and location of the implant in the jaw, namely in the premolar or molar region.

When it comes to age, it was observed that older people (≥41 years of age) had a higher estimated MBL than younger patients. One possible explanation for this is that bone mass density decreases with aging, and it may sometimes be due to age-related osteoporosis [24,25]. Negri et al. [26] investigated the impact of age, sex and insertion site on MBL. The results showed a difference in MBL between the mandible and the maxilla in patients with different age groups, namely <50 years, 50–60 years and >60 years of age. A higher MBL was observed among the elderly patients in the maxilla, while no difference in MBL was seen between the different ages in the mandible. The mandible mostly consists of cortical bone, and the maxilla consists of trabecular bone [27]. Age-related bone loss is more common in trabecular bone, which explains the difference in marginal bone loss between the maxilla and the mandible observed in the study [26].

Regarding bruxism, higher MBL was observed around implants placed in patients who had the condition in comparison to non-bruxers. Bruxism has been associated with a higher prevalence of dental prosthetic technical complications [12,28,29,30,31,32], increased risk of implant failure [12,33,34,35], and implant fracture [36]. The lower tactile sensitivity around implants in relation to natural teeth may increase the risk of higher loads being applied to implant-supported restorations during bruxism due to the limited proprioceptive feedback [37,38], making them more prone to occlusal overload and possible subsequent greater MBL. This could be possible, even though the group of implants in probable bruxers consisted of far fewer samples than in the groups of non-bruxer patients; more balanced groups would be necessary to give more reliability to these results concerning bruxism. The results of the first clinical study comparing MBL in a group of bruxers in relation to a matched group of non-bruxers suggested that bruxism increases the risk of MBL over time [39].

The results of our study also suggested that the tooth region had a statistically significant influence on MBL over time, namely greater estimated MBL around implants in the premolar than in the molar region. Data from the literature could suggest that the opposite would be expected. Shinogaya et al. [40] observed that the occlusal load center was located at the distal aspect of the first upper molar. Moreover, D’amico et al. [41] observed that the masticatory forces were higher in the posterior regions of the oral cavity. Here the very unbalanced sample sizes regarding groups of implant locations in the jaws could be a possible reason for these discrepant results. There were far more implants placed in the premolar area than in the molar area, and unequal sample sizes and variances dramatically affect statistical power and type I error rates [42]. One could argue that the number of implants in bruxers and in non-bruxers was also very unbalanced. However, in the case of bruxism, previous evidence gives some support to what was observed, whereas when it comes to implants in different anterior-posterior locations of the jaws, the expected result is just the opposite of what the present results suggested.

As for limitations in the present study, this was a dental-record based retrospective study. The nature of a retrospective study inherently results in flaws. These problems were manifested by the gaps in information and incomplete records. Furthermore, all data rely on the accuracy of the original examination and documentation. Items may have been excluded in the initial examination or not recorded in the dental chart. Moreover, although patients with a history or with signs of periodontal disease were treated in a different department of the aforementioned clinic and therefore not included in the present study, this does not preclude that some of the included patients may later develop peri-implantitis.

## 5. Conclusions

In conclusion, the results of the present study suggest that differences in implant inclination, anatomical and clinical CIR do not have a clear influence on MBL over time in implants supporting single crowns in the posterior region of the jaws.

## Figures and Tables

**Table 1:** Descriptive data of the implants and their single crowns (cases) included in the study, with follow-up time between the different factors. The statistical unit is the implant, not the patient.

Factor	Number of Cases (%)	Clinical Follow-Up (Months) Mean ± SD (Min–Max)	*p* Value ^a^	Radiological Follow-Up (Months) Mean ± SD (Min–Max)	*p* Value ^a^
**Sex**					
Male	118 (37.3)	120.0 ± 65.3 (18.2–323.3)	0.481	107.1 ± 62.8 (36.4–319.5)	0.452
Female	198 (62.7)	116.4 ± 64.8 (36.1–361.1)		103.5 ± 65.0 (36.1–358.7)	
**Age (years)**					
<20	95 (30.1)	123.3 ± 65.6 (18.2–361.1)	0.056	116.3 ± 63.8 (40.8–358.7)	0.042
20–40	113 (35.7)	128.0 ± 76.8 (36.1–346.3)		114.0 ± 77.5 (36.1–337.7)	
>40	108 (34.2)	102.1 ± 45.3 (33.6–248.9)		89.3 ± 43.0 (36.4–225.1)	
**Jaw**					
Maxilla	145 (45.9)	118.5 ± 67.1 (36.1–346.3)	0.848	102.3 ± 66.1 (36.1–337.7)	0.201
Mandible	171 (54.1)	117.1 ± 63.1 (18.2–361.1)		106.9 ± 62.5 (36.7–358.7)	
**Region**					
Premolar	294 (93.0)	116.5 ± 64.1 (18.2–361.1)	0.260	103.2 ± 63.0 (36.1–358.7)	0.155
Molar	22 (7.0)	135.2 ± 73.7 (43.5–296.8)		126.2 ± 76.1 (43.5–296.0)	
**Crown material** ^b^					
Metal ceramic	144 (47.2)	112.6 ± 64.1 (33.6–346.3)	<0.001	100.3 ± 62.3 (36.7–337.7)	<0.001
Full ceramic	101 (33.1)	141.1 ± 69.0 (18.2–320.6)		128.5 ± 68.6 (38.3–319.5)	
Zirconia	54 (17.7)	86.6 ± 34.9 (36.1–181.4)		72.5 ± 31.6 (36.1–176.8)	
Metal acrylic	6 (2.0)	175.4 ± 103.1 (112.0–361.1)		162.5 ± 111.7 (82.2–358.7)	
**CIR (anatomical)**					
≤0.66	107 (33.9)	130.3 ± 67.5 (18.2–344.8)	0.002	115.7 ± 66.5 (38.1–319.5)	0.008
0.67–0.82	105 (33.2)	120.3 ± 67.9 (33.6–361.1)		107.8 ± 68.6 (38.0–358.7)	
≥0.83	104 (32.9)	102.3 ± 55.8 (36.1–323.3)		90.6 ± 54.1 (36.1–310.5)	
**CIR (clinical)**					
≤0.95	107 (33.8)	125.0 ± 74.6 (43.5–361.1)	0.932	109.6 ± 74.5 (36.4–358.7)	0.756
0.96–1.15	101 (32.0)	114.5 ± 61.3 (18.2–346.3)		100.6 ± 58.8 (36.1–337.7)	
≥1.16	108 (34.2)	113.7 ± 57.4 (36.7–343.3)		104.0 ± 57.6 (36.7–328.7)	
**Implant inclination**					
<78	104 (32.9)	117.4 ± 62.7 (36.1–346.3)	0.601	101.5 ± 62.7 (36.1–337.7)	0.445
78–84	108 (34.2)	116.5 ± 69.0 (18.2–343.3)		104.6 ± 67.6 (36.1–328.7)	
84–90	104 (32.9)	119.4 ± 63.2 (33.6–361.1)		108.3 ± 62.1 (36.7–108.3)	
**Implant diameter**					
3.00–3.50	31 (9.8)	110.5 ± 60.6 (36.1–243.7)	0.093	94.5 ± 57.7 (36.1–242.8)	0.077
3.75–4.10	250 (79.1)	121.4 ± 67.1 (18.2–361.1)		108.9 ± 66.8 (36.1–358.7)	
4.30–5.00	35 (11.1)	98.3 ± 47.2 (44.9–193.4)		84.8 ± 42.5 (36.4–167.7)	
**Prosthesis fixation** ^b^					
Cemented	183 (60.0)	127.2 ± 62.0 (18.2–344.8)	<0.001	111.9 ± 58.5 (36.1–319.5)	<0.001
Screwed	122 (40.0)	104.5 ± 69.5 (36.1–361.1)		95.5 ± 72.7 (36.1–358.7)	
**Bruxism** ^b^					
No	253 (91.7)	119.0 ± 63.1 (18.2–361.1)	0.253	105.0 ± 62.4 (36.1–358.7)	0.294
Yes	23 (8.3)	113.1 ± 74.8 (50.9–344.8)		99.1 ± 67.9 (36.4–292.1)	
**Smoking** ^b^					
No	215 (78.5)	117.3 ± 61.0 (18.2–361.1)	0.884	103.2 ± 59.1 (36.1–358.7)	0.965
Yes ^c^	59 (21.5)	123.6 ± 75.6 (47.8–346.3)		110.0 ± 75.8 (39.6–337.7)	

CIR—Crown-to-implant ratio. ^a^ Comparison of the mean follow-up time between the groups of each variable (Mann—Whitney test for 2 groups, Kruskal-Wallis test for 3 or more groups). ^b^ For the cases with available information. ^c^ It includes 4 cases of former smokers.

**Table 2:** Univariate linear regression analysis for MBL.

Factor	Linear Equation *	*p* Value ^a^	R^2^ Linear
**Sex**			
Male	y = −0.27 − 0.00142x	<0.001	0.014
Female	y = −0.27 − 0.00129x		0.010
**Age (years)**			
<20	y = −0.11 − 0.00166x	<0.001	0.024
20–40	y = −0.23 − 0.00015x		<0.001
>40	y = −0.32 − 0.00542x		0.104
**Jaw**			
Maxilla	y = −0.33 − 0.00130x	0.159	0.011
Mandible	y = −0.21 − 0.00141x		0.013
**Tooth region**			
Premolar	y = −0.26 − 0.00166x	0.006	0.018
Molar	y = −0.36 + 0.00224x		0.031
**Crown material** ^b^			
Metal ceramic	y = −0.27 − 0.00178x	0.089	0.008
Full ceramic	y = −0.30 − 0.00208x		0.007
Zirconia	y = −0.14 − 0.00232x		0.065
Metal acrylic	y = −0.22 − 0.00140x		0.019
**CIR (anatomical)**			
≤0.66	y = −0.19 − 0.00105x	<0.001	0.009
0.67–0.82	y = −0.29 − 0.00117x		0.010
≥0.83	y = −0.30 − 0.00261x		0.029
**CIR (clinical)**			
≤0.95	y = −0.27 − 0.00084x	<0.001	0.006
0.96–1.15	y = −0.24 − 0.00095x		0.006
≥1.16	y = −0.29 − 0.00223x		0.027
**Implant inclination**			
<78	y = −0.24 − 0.00108x	<0.001	0.007
78–84	y = −0.29 − 0.00148x		0.015
84–90	y = −0.27 − 0.00137x		0.012
**Implant diameter**			
3.00–3.50	y = −0.08 − 0.00153x	<0.001	0.014
3.75–4.10	y = −0.29 − 0.00128x		0.011
4.30–5.00	y = −0.24 − 0.00160x		0.011
**Prosthesis fixation** ^b^			
Cemented	y = −0.25 − 0.00164x	<0.001	0.014
Screwed	y = −0.30 − 0.00083x		0.006
**Bruxism** ^b^			
No	y = −0.27 − 0.00103x	<0.001	0.007
Yes	y = −0.27 − 0.00480x		0.118
**Smoking** ^b^			
No	y = −0.31 − 0.00150x	0.611	0.012
Yes ^c^	y = −0.18 − 0.00097x		0.010

CIR—Crown-to-implant ratio. * For the linear equation, “x” represents the number of months. ^a^ Comparison of the slope of the equation (variation of MBL in mm in time) between groups. ^b^ For the cases with available information. ^c^ It includes 4 cases of former smokers.

**Table 3:** Linear mixed-effects model for MBL.

Predictor Variables	F Statistic	*p* Value
Sex	0.079	0.778
Age	48.457	<0.001
Tooth region	11.898	0.001
CIR (anatomical)	0.468	0.494
CIR (clinical)	0.717	0.397
Implant inclination	0.414	0.520
Implant diameter	3.453	0.063
Prosthesis fixation	3.552	0.060
Bruxism	10.645	0.001

## Data Availability

Restrictions apply to the availability of these data. Data were obtained from patients treated at Folktandvården Skåne AB, Malmö, Sweden, and cannot be shared, in accordance with the General Data Protection Regulation (EU) 2016/679.
